# Physiological Stress in Safer Cycling in Older Age (SiFAr-Stress): A Randomized Controlled Trial

**DOI:** 10.1177/07334648251316950

**Published:** 2025-02-26

**Authors:** Linda Becker, Cornel Sieber, Nicolas Rohleder, Ellen Freiberger, Robert Kob, Sabine Britting

**Affiliations:** 1 9171 Friedrich-Alexander-Universität Erlangen-Nürnberg, Nürnberg, Germany; 2 Kantonsspital Winterthur, Winterthur, Switzerland

**Keywords:** community-dwelling, aging, cycling, stress, cortisol, C-reactive protein

## Abstract

One possibility for maintaining mobility in older age is cycling. We investigated the impact of the multicomponent “*Safer Cycling in Older Age*” (SiFAr) intervention on psychological and physiological stress. Participants were 98 community-dwelling older adults (73.4 ± 5.4 years). Bedtime cortisol, hair cortisol concentrations, and C-reactive protein were measured before and after the 8-week SiFAr intervention and at follow-up. Additionally, acute stress responses were assessed during the second and seventh training sessions using salivary alpha-amylase and cortisol assessments. We found a decrease in acute perceived stress, anxiety, fear of falling, and uncertainty during the cycling trainings. Moreover, long-term perceived stress significantly decreased. No significant changes were found for any of the physiological stress measures. We conclude that cycling had a positive impact on perceived stress and wellbeing. Further research with more intense trainings is needed to fully understand the associations between cycling in older age and physiological stress.

## Highlights

**What this paper adds**

• Psychological and physiological stress during cycling was assessed in a community-dwelling sample.
• Psychological stress, anxiety, uncertainty, and fear of falling decreased during the cycling trainings.
• Perceived stress decreased in the long-term.

**Applications of study findings**

• Cycling interventions can reduce stress in community-dwelling older adults.
• An 8-week multicomponent training such as SiFAr can improve cycling competence.
• Cycling is a recommended means of transportation for community-dwelling older adults.

## Introduction

Promoting health and wellbeing in older age is fundamental. One important factor that promotes health-related quality of life in older age is mobility (Davis et al., 2015). Especially in community-dwelling older adults, cycling as an environmentally friendly form of everyday transportation has become increasingly popular. Since the introduction of e-bikes, even more people benefit from cycling and its impact on health, wellbeing, and independency (Oja et al., 2011). Health benefits of cycling are manifold and have been found across the lifespan (Bauman & Rissel, 2009). Cycling is positively associated with physical fitness, cardiovascular health, physical performance, and metabolic processes (Bourne et al., 2018; Oja et al., 2011).

However, there are also concerns, which must be considered. Most importantly, an inappropriate behavior of cyclists in combination with significant increase in traffic volume can promote an increasing number of accidents of older cyclists (Levulytė et al., 2016). Cycling-related concerns such as not feeling safe due to road traffic, uncertainties related with the change to motorized bicycles, or a lack of practice can be perceived as frightening or stressful and can lead to not using the bicycle or only under uncertainty. One important factor in older cyclists is fear of falling (FoF; Jørstad et al., 2005). Generally, FoF (investigated in the context of fall prevention) is associated with several health outcomes such as depression, the number of social contacts, and quality of life (Schoene et al., 2019).

If cycling-related concerns are not addressed appropriately, cycling may be a significant stressor, especially for older cyclists. In situations associated with stress, various physiological signal cascades are initiated. The first, immediate response is the activation of the sympathetic nervous system (SNS), which is associated with the fight-or-flight response (Ulrich-Lai & Herman, 2009). This leads to the release of the catecholamines adrenaline and noradrenaline, an increase in heart rate and blood pressure, and further responses. The second physiological stress response is the activation of the hypothalamic–pituitary–adrenal (HPA) axis, which initiates the release of glucocorticoids (i.e., cortisol in humans) from the adrenal cortex (Ulrich-Lai & Herman, 2009). HPA-axis responses are slower than SNS responses. Cortisol peaks have been found around 20 minutes after the end of the stressor in socially evaluative situations (Becker & Rohleder, 2019; Liu et al., 2017). For pure physical stressors of medium or low intensity such as running, cycling, or strength training only SNS, but no HPA axis, responses have been reported (Becker, Semmlinger, & Rohleder, 2021; Kindermann et al., 1982). However, for stressors which are associated with anxiety or uncertainty both, SNS as well as HPA-axis responses can be expected. With a further delay of several hours after the onset of the stressor, complex responses of the immune system have been found such as an increase of pro-inflammatory cytokines (e.g., interleukin-6 and C-reactive protein [CRP], Marsland et al., 2017).

In the long term, especially when people not adequately cope with stressors, chronic stress can occur. Chronic stress is one major risk factor for chronic diseases such as hypertension, cardiovascular diseases, diabetes mellitus, or stroke (Chrousos, 2009; McEwen, 2017). Basal activity of the SNS and the HPA axis can be altered in the long term, which translates to downstream changes in the inflammatory system, in the end contributing to the development of low-grade inflammation and, thus, an increase in peripheral inflammatory activity (Rohleder et al., 2009). Chronic low-grade inflammation is one key factor for morbidity, mortality, and a predictor for many age-related diseases (Harris et al., 1999). Therefore, cycling-related stress as well as further concerns regarding cycling such as FoF during cycling and in general may in the end be negatively associated with peoples’ health and wellbeing.

To date, studies are missing, in which the associations between cycling-related concerns and physiological stress have been investigated. The goal of our study “*Physiological Stress in Safer Cycling in Older Age*” (SiFAr-Stress; Britting et al., 2021) was to investigate psychological and physiological stress in the context of the multicomponent cycling intervention SiFAr. In SiFAr, older people with self-perceived needs for improvement in cycling performance participated in a multicomponent program, which included several exercises (e.g., cycling techniques, balance and strength exercises with and without the bicycle). The SiFAr trial led to a significant improvement in cycling skills in the intervention group in comparison to the control group (Keppner, Krumpoch, et al., 2023). In SiFAr-Stress, additional parameters associated with physiological and perceived stress were collected beside the main outcomes. We expected acute psychological (e.g., changes in perceived stress) as well as physiological responses of the SNS during the cycling sessions. SNS activity was assessed using salivary alpha-amylase (sAA; Nater & Rohleder, 2009). Furthermore, we hypothesized that “*… salivary cortisol levels […] will be lower after the seventh than after the second lesson*” (Britting et al., 2021; p. 4). Beside markers for acute stress responses, chronic stress measures were assessed such as bedtime cortisol, hair cortisol concentration (HCC), and CRP levels. We hypothesized that the SiFAr intervention will lead to general health-promoting effects in the long term, that is, a reduction in chronic stress-related parameters in the intervention group (IG) in comparison to the control group (CG).

## Methods

### Participants

**Figure 1.**
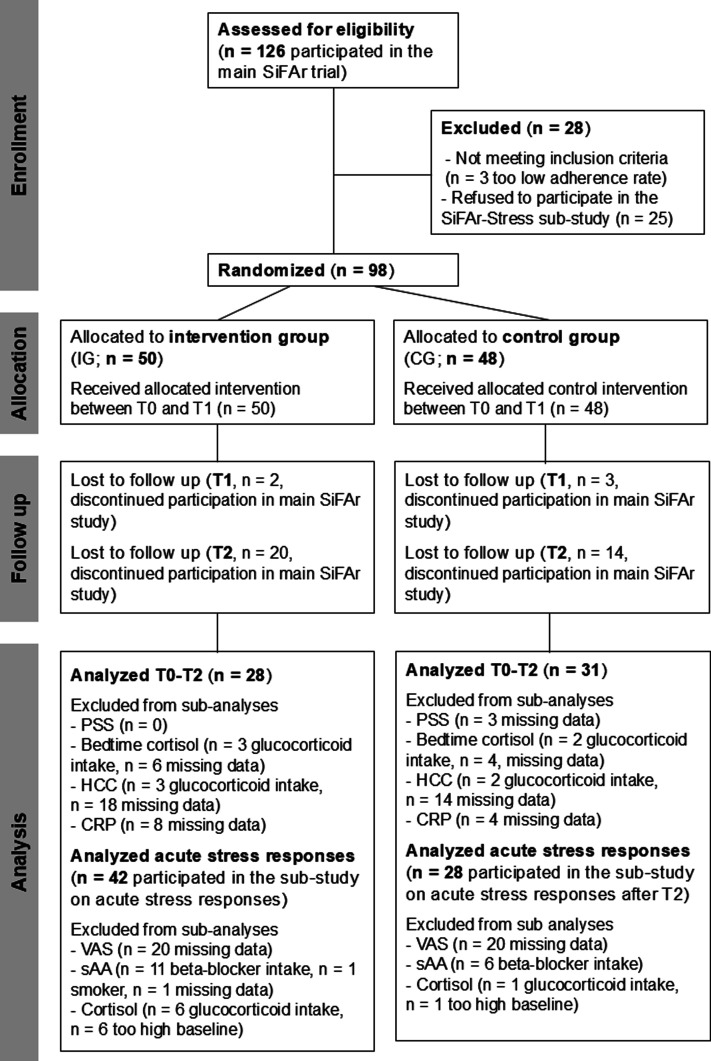
Consolidated Standards of Reporting Trials (CONSORT) flowchart diagram. Note. PSS = Perceived Stress Scale (Cohen et al., 1994; Klein et al., 2016); CRP = C-reactive protein, HCC = hair cortisol concentration; sAA = salivary alpha-amylase, VAS = visual-analogue scale.

Overall, *N* = 126 participants took part in the main SiFAr study (Keppner, Krumpoch, et al., 2023; Keppner, Sieber, et al., 2023) of which *N* = 101 were willing to additionally join the sub-study SiFAr-Stress. Three of these participants had to be excluded from statistical analysis due to a too low adherence rate (less than 6 out of 8 training sessions). This resulted in a final sample size of *N* = 98 (73.4 ± 5.4 years; *n* = 61 [62.2%] female; BMI = 26.3 ± 4.1 kg/m^2^) for SiFAr-Stress. Forty-three of these participants (43.9%) were using e-bikes. Participants were allowed to decide for each physiological measure individually whether they wanted to participate. Some samples had to be excluded from further analyses due to exclusion criteria (e.g., drug intake) as specified below. The final sample sizes for each measure are provided in the Consolidated Standards of Reporting Trials (CONSORT) diagram in Figure 1.

The SiFAr trial has been prospectively registered in April 2020 at clinicaltrials.gov (no.: NCT04362514). The study protocol of SiFAr-Stress has also been published prospectively (Britting et al., 2021). A power analysis has been conducted, which suggested an optimal sample size of *N* = 111 participants for SiFAr-Stress. Therefore, we were not able to fully meet this requirement. The study was approved by the local ethics committee of the Friedrich-Alexander-Universität Erlangen-Nürnberg (FAU; no.: 22_20B). Written and informed consent was provided by all participants. The study was conducted in accordance with the Helsinki Declaration.

### Design and Setting of the Study

**Figure 2.**
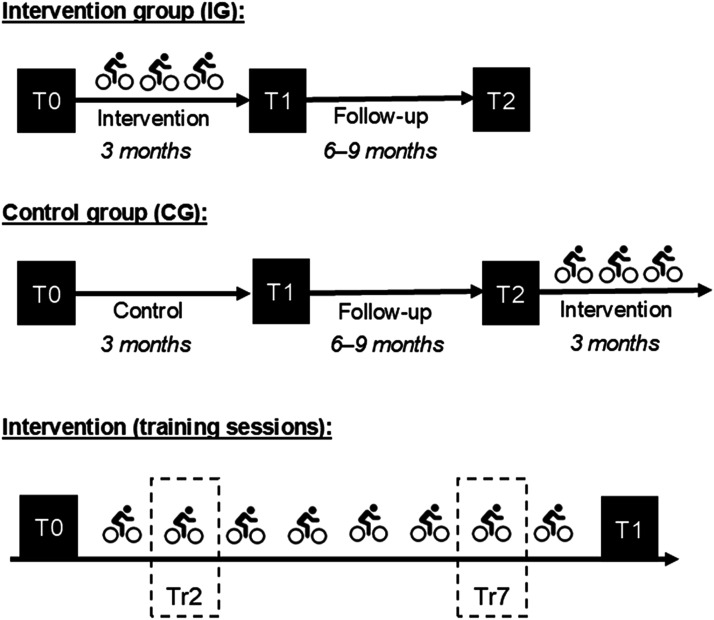
Study design. All main outcome variables were collected at all measurement time points T0, T1, and T2. The intervention consisted of eight weekly training sessions. Measures for investigation of acute effects were collected during training 2 (Tr2) and training 7 (Tr7).

SiFAr-Stress is a sub-study of SiFAr, which is a randomized controlled trial with a parallel group design and three measurement time points T0, T1, and T2 (Figure 2). SiFAr addresses the improvement of cycling competence in older people. Data was collected between July 2020 and May 2022 for SiFAr-Stress. Location of data collection and the training sessions was Nuremberg or Erlangen (Bavaria, Germany).

### Procedure

For those participants who agreed to participate in the SiFAr-Stress sub-study, bedtime cortisol, HCC, and CRP samples as well as perceived chronic stress were additionally collected beside the main outcomes of SiFAr at T0, T1, and T2. Furthermore, participants provided three saliva samples during the second and seventh training sessions (Tr2 and Tr7) as well as self-reports on acute perceived stress, anxiety, FoF, and uncertainty.

Participants were randomly assigned to the IG or CG. Allocation was stratified by sex and bicycle type (e-bike or unmotorized bicycle). The IG received the cycling intervention between T0 and T1. The training sessions included cycling short distances, balancing, and braking training. It also included fitness exercises aimed at training the lower extremities, which were performed without using the bike. Further details of the cycling intervention are provided in Siebentritt et al. (2021). During this time interval, the CG received three health-related leaflets (one per month) covering the following topics: physiological changes with age, safety check of bicycle, and traffic regulations as control intervention. After completion of T2, the CG had the possibility to join the cycling intervention. At T0, T1, and T2, all participants took part in a comprehensive assessment at the Institute of Biomedical Aging (Nuremberg, Germany), including questionnaires, functional and cognitive testing, as well as the collection of capillary blood and hair samples. Additionally, bedtime saliva samples were provided on two consecutive days in the week after the assessments at the participants’ homes. Moreover, participants took part in a cycling course at all three time points to assess their cycling-related skills (Siebentritt et al., 2021).

The cycling intervention included eight weekly sessions, each lasting about 60 minutes, and was conducted in small groups of about seven participants. At Tr2 and Tr7, saliva samples were collected from the participants of the SiFAr sub-study at three time points (before, immediately after, and 20 minutes after the intervention; Figure 2). Visual-analogue scales (VASs) were applied to assess currently perceived stress, anxiety, uncertainty, and FoF during the cycling trainings.

### Inclusion and Exclusion Criteria

A full list of inclusion and exclusion criteria is provided in the study protocol (Britting et al., 2021). In short, participants aged 65 years or older, were beginners with the e-bike, reported uncertainties while cycling, or were re-entrants in cycling. Self-perceived need for improvement in cycling was identified during a selection interview using the question “*Do you feel less safe when cycling than before?*”. Reasons for exclusion were diseases or certain factors that contradicted safe participation in the intervention. Furthermore, participants were partially excluded from analyses of physiological stress markers if they reported intake of beta-blocker (excluded for SNS analyses) or glucocorticoids (exclusion for HPA-axis analyses) or were heavy smokers (> 5 cigarettes a day, exclusion for SNS analyses).

### Materials

#### Physiological Stress Assessment

For assessment of acute physiological stress responses during Tr2 and Tr7, saliva samples were collected using Salivettes (Sarstedt, Nümbrecht, Germany). Participants were instructed to keep the Salivettes in their mouths for at least 1 minute, to move it back and forth, but not to chew on it. Samples were stored at −30°C until laboratory analysis.

For assessment of bedtime cortisol levels, Salivettes were used as well. Participants were instructed to take the samples at bedtime before brushing their teeth and at least 1 hour after dinner. Salivettes were stored in participants’ fridge until they were sent via mail to the laboratory the next day, where they were stored at −30°C.

For assessment of HCC as long-term measure of HPA-axis activity, participants provided hair samples during the assessments at T0, T1, and T2. Hair samples were stored in a cool room and protected from light until analysis.

Blood samples were collected by means of dried blood spots (DBSs; McDade et al., 2007) during the assessments, from which CRP levels were determined afterward. DBS samples were dried overnight for at least 8 hours in a darkened room and were frozen the next morning in airtight envelopes containing silicate.

#### Stress Rating Scales

For assessment of acute perceived stress, anxiety, uncertainty, and FoF during the training sessions, established VAS with 10-point Likert scales were used (Becker et al., 2022).

For assessment of perceived stress at the moment of bedtime saliva sampling and during the sampling day, 6-point smiley rating scales were applied, which have also been used previously (Pretscher et al., 2021).

#### Questionnaires

Demographic variables (age, sex, BMI, years of education, and living situation), medication intake (beta-blockers, glucocorticoids, and further drugs), as well as psychological variables (perceived stress during the last month, depression, and general FoF) were collected using standardized questionnaires, which were filled out by the participants during the assessments. Perceived stress during the last months was assessed using a German translation of the 10-item version of the Perceived Stress Scale (PSS; Cohen et al., 1994; Klein et al., 2016). General FoF was assessed by means of the German version of the Falls Efficacy Scale-International Version (FES-I; Delbaere et al., 2010; Dias et al., 2006). Depression was assessed using the Geriatric Depression Scale (GDS; Sheikh & Yesavage, 1986). Health-related quality of life was rated on the VAS from the EQ-5D (range 0–100; Lutomski et al., 2017).

#### Assessment of Cycling and Physical Performance

At each measurement time point, participants conducted a cycling course. The course included seven tasks (slalom, slow cycling, dismounting into a hula hoop, getting on the bicycle, cycling through a narrow alley, turning to the off-side, and precise braking). The number of errors was considered as measure for cycling performance.

General physical performance was assessed using the Short Physical Performance Battery (SPPB; Freiberger et al., 2012; Pavasini et al., 2016). The SPPB assesses a set of functional tasks related to the lower extremities such as static balance tests and repeatedly getting up from a chair.

#### Cognitive Abilities

A screening for mild cognitive impairment (MCI) was conducted using the Montreal Cognitive Assessment (MoCA; Nasreddine et al., 2005). Sum scores of 25 and lower were used as indicators for MCI.

### Laboratory Analyses

Analyses of saliva and DBS samples were conducted in the laboratory of the Chair of Health Psychology at FAU by trained staff. Salivettes were thawed and centrifuged at 2,000 g at 4°C on analysis day. Concentrations of sAA were measured using an enzyme kinetic assay. For salivary cortisol assessment, high-sensitivity luminescence immunoassay (IBL international, Hamburg, Germany) was used. Both, sAA and cortisol analyses were conducted in duplicates. Intra-assay coefficients of variance were below 10% for both, sAA and cortisol.

On the day before CRP level estimation, a circle with a diameter of 3.5 mm was punched out from the DBS samples and was eluted overnight in phosphate buffered saline which contained 0.1% Tween 20 solution. Samples were shaken at 300 rpm for 1 hour before further processing the next morning. For the CRP assessment, high-sensitive enzyme-linked immunosorbent assays were used. Intra-assay coefficients of variance were below 5% for CRP.

Hair samples were sent to Dresden LABservice GmbH (Dresden, Germany), where HCC was determined from the youngest (i.e., closest to the scalp) 3 cm by means of coupled tandem mass spectrometry. Intra-assay coefficients of variance were below 10% for HCC.

### Statistical Analyses

For statistical analyses, JASP (version 0.18.0.0 for Windows) was used. Descriptive statistics, including frequencies, means (*M*), and standard deviations (*SD*) were computed to describe the sample characteristics. Because of skewness, all sAA, cortisol, and CRP levels were transformed using the natural logarithm (ln). Potential differences in socio-demographic variables between the groups were tested using Chi^2^ tests for nominal variables and independent samples *t*-tests for metric variables. For hypotheses testing and evaluation of the intervention in general, analyses of variance (ANOVAs) for repeated measurements with the between-subjects factors “group” (IG vs. CG) and the within-subjects factor “time” (T0, T1, and T2) were calculated. For analysis of acute effects during the training sessions, the within-subjects factors “time” (pre, post, and +20 min) and “training” (Tr2 vs. Tr7) were considered. If necessary, sphericity violations, which were determined by Mauchly’s test of sphericity, were corrected by adjusting the degrees of freedom with the procedure by Greenhouse and Geisser. Partial eta-squares η_p_^2^ were used as measures for effect sizes for ANOVAs. For post-hoc comparisons, *t-*tests for paired samples, *t*-tests for independent samples, and Pearson’s correlations were used. Cohen’s *d* was used as measure for effect size for *t*-tests. The potential confounders age, sex, and BMI were included as covariates in the statistical analyses for all physiological variables. An α-level of α = .05 was used for all statistical analyses as was specified in the study protocol.

## Results

### Descriptive Statistics

An overview about participants’ socio-demographic and psychological variables at baseline is provided in Tables 1 and 2. There were no differences between the CG and the IG in any of these variables (i.e., age, sex, BMI, education, bicycle type, MCI, GDS, MoCA, FES-I, SPPB, EQ-5D, number of errors in the course, and living situation; all *p* ≥ .105).

**Table 1.** Participant Characteristics at Baseline (T0) for Metric Variables.

	Overall			IG			CG		
Variable	*M*	*SD*	*N*	*M*	*SD*	*N*	*M*	*SD*	*N*
Age (years)	73.4	5.4	98	72.5	5.0	50	74.3	5.6	48
BMI (kg/m^2^)	26.3	4.0	98	26.6	4.2	50	25.9	3.9	48
Education (years)	15.6	3.1	97	14.6	3.0	50	14.5	3.3	47
GDS score	1.2	1.4	94	1.2	1.2	50	1.3	1.6	44
MoCA score	26.5	2.3	97	26.6	2.2	50	26.4	2.4	47
FES-I score	8.0	1.7	97	7.8	1.8	50	8.2	1.7	47
SPPB score	11.1	1.2	98	11.3	0.8	50	10.9	1.4	48
Number of errors in the course	9.3	7.4	97	9.3	6.7	50	9.4	8.1	47
Health-related quality of life (VAS)	78.1	11.0	97	79.7	9.5	50	76.3	12.2	47

Note. IG = intervention group, CG = control group, *M* = mean, *SD* = standard deviation, BMI = body mass index, GDS = Geriatric Depression Scale (Sheikh & Yesavage, 1986), MoCA = Montreal Cognitive Assessment (Nasreddine et al., 2005), FES-I = Falls Efficacy Scale-International Version (Delbaere et al., 2010; Dias et al., 2006), SPPB = Short Physical Performance Battery (Freiberger et al., 2012; Pavasini et al., 2016); VAS = visual-analogue scale (range 0–100).

**Table 2.** Participant Characteristics for Nominal-Scaled Variables.

		Overall			IG			CG		
Variable	Values	*n*	*N*	%	*n*	*N*	%	*n*	*N*	%
Sex	Female	61	98	62.2	34	50	68.0	27	48	56.3
	Male	37	98	37.8	16	50	32.0	21	48	43.8
Type of bicycle	e-bike	43	98	43.9	25	50	50.0	18	48	37.5
	Unmotorized bicycle	48	98	49.0	23	50	46.0	25	48	52.1
	Missing	7	98	7.1	2	50	4.0	5	48	10.4
Living condition	Living alone	38	98	38.8	22	50	44.0	16	48	33.3
	Living with spouse	55	98	56.1	26	50	52.0	29	48	60.4
	Living with children	4	98	4.1	3	50	6.0	1	48	2.1
	Living with a friend	3	98	3.1	2	50	4.0	1	48	2.1
	Other living condition	1	98	1.0	0	50	0.0	1	48	2.1
	Missing	1	98	1.0	0	50	0.0	1	48	2.1
MCI at T0	Yes	32	98	32.7	17	50	34.0	15	48	31.3
	No	66	98	67.3	33	50	66.0	33	48	68.8

Note. IG = intervention group, CG = control group, *n* = count, *N* = overall number, MCI = mild cognitive impairment.

There were also no baseline differences between IG and CG for the main outcome variables (i.e., PSS, HCC, bedtime cortisol, and CRP; all *p* ≥ .226).

### General Evaluation of the Intervention

**Figure 3.**
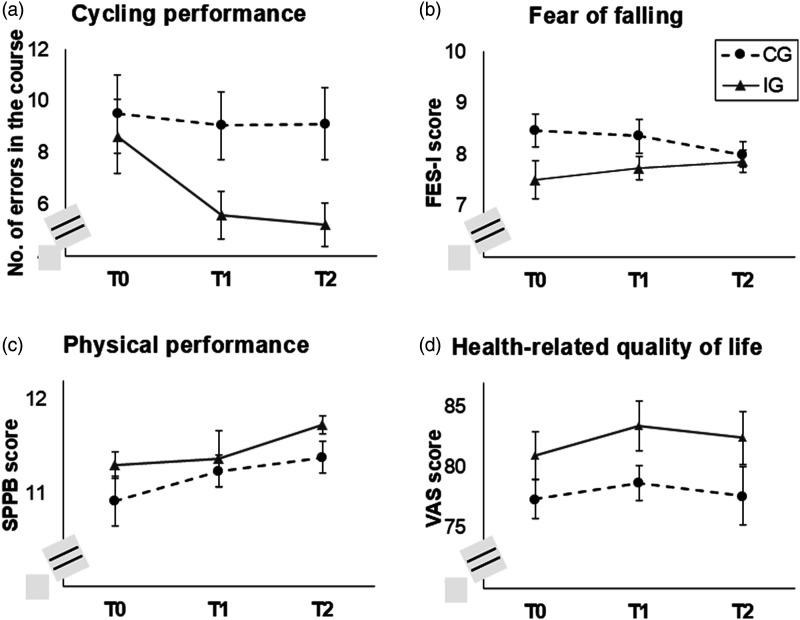
Evaluation of the cycling intervention for our sub-sample (*N* = 98): (a) cycling performance in the cycling course, (b) general fear of falling, and (c) general physical performance. Note. FES-I = Falls Efficacy Scale-International Version (Delbaere et al., 2010; Dias et al., 2006); SPPB = Short Physical Performance Battery (Freiberger et al., 2012; Pavasini et al., 2016); VAS = visual-analogue scale (range 1–100).

For cycling performance, a significant main effect of the factor time (*F*(1.70) = 5.53, *p* = .008, η_p_^2^ = 0.09) and a significant interaction time*group (*F*(1.66) = 3.34, *p* = .047, η_p_^2^ = 0.06) were found. The number of errors in the cycling course significantly changed between T0 and T1 in the IG (*t*(26) = 3.05, *p* = .005, *d* = 0.59) but did not change in the CG (*p* = .867; Figure 3(a)). Therefore, the cycling intervention was successful.

For general FoF, no significant effects were observed (all *p* ≥ .098; Figure 3(b)). For general physical performance, a significant main effect of the factor time was found (*F*(1.72) = 3.69, *p* = .034, η_p_^2^ = 0.06), indicating an overall increase between T0 and T2 (*t*(63) = 3.30, *p* = .002, *d* = 0.41; Figure 3(c)). For health-related quality of life, no significant effects were found (all *p* ≥ .077); Figure 3(d)).

### Acute Stress Responses

**Figure 4.**
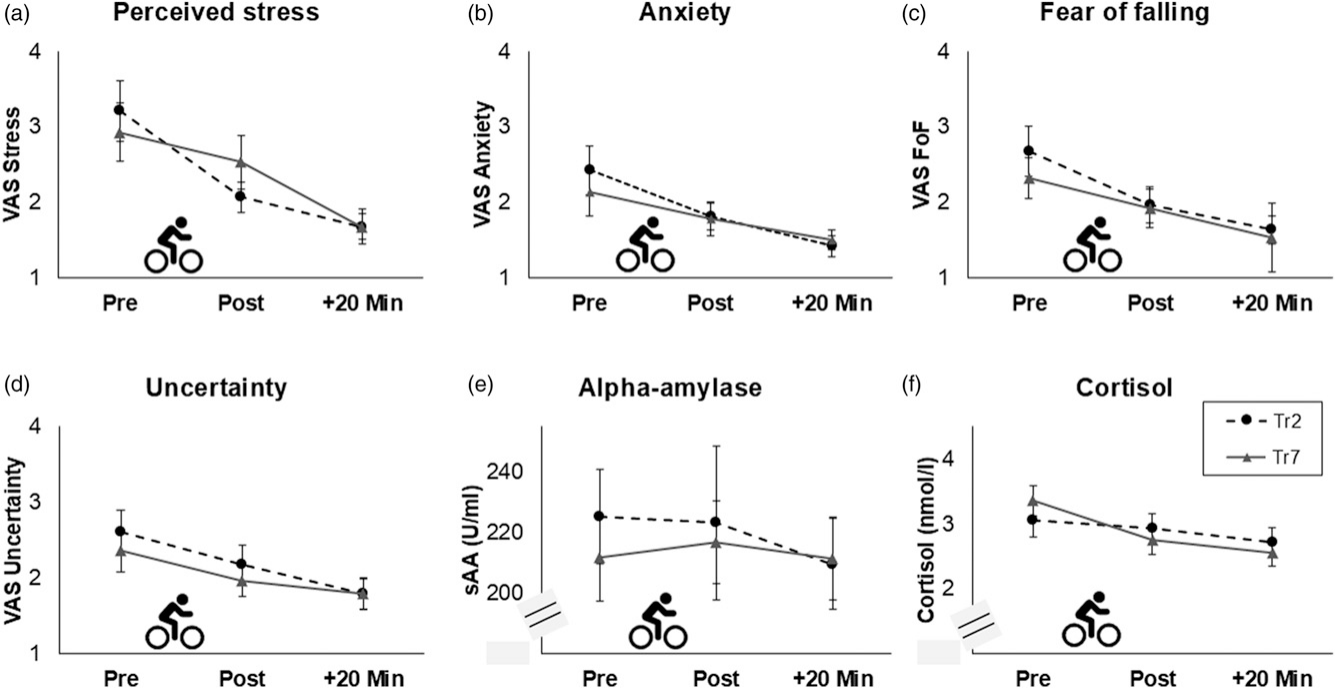
Acute effects of the cycling trainings on (a) perceived stress, (b) anxiety, (c) fear of falling (FoF), (d) insecurity, (e) salivary alpha-amylase (sAA), and (f) cortisol for the second (Tr2) and seventh (Tr7) training sessions. Standard errors are shown as error bars. sAA and cortisol levels were transformed using the natural logarithm for statistical analyses. Note. VAS = visual-analogue scale (range: 1–10).

For perceived stress, a main effect of the factor time was found (*F*(1.47) = 22.48, *p* < .001, η_p_^2^ = 0.45), indicating a decrease in perceived stress during both, Tr2 (e.g., pre-post2: *t*(27) = 3.53, *p* = .002, *d* = 0.67) and Tr7 (e.g., pre-post2: *t*(28) = 3.53, *p* = .001, *d* = 0.66). Similar patterns (i.e., main effects of time and an overall decrease) were found for perceived anxiety (*F*(1.26) = 9.56, *p* = .002, η_p_^2^ = 0.26), acute FoF (*F*(1.41) = 16.58, *p* < .001, η_p_^2^ = 0.38), and uncertainty (*F*(1.58) = 9.44, *p* < .001, η_p_^2^ = 0.26; Figure 4(a)–(d)).

For sAA as measure for SNS activity, no significant effects were found (all *p* ≥ .182). Therefore, sAA levels did not significantly change during the training sessions (Figure 4(e)).

For cortisol as measure for HPA-axis activity, a significant main effect of the factor training was found (*F*(1) = 5.85, *p* = .019, η_p_^2^ = 0.10), reflecting a tendency to higher cortisol baseline levels (i.e., before the cycling training) in Tr7 than in Tr2 (*t*(56) = 1.98, *p* = .053, *d* = 0.26). The interaction time*training slightly missed statistical significance (*F*(1.54) = 2.81, *p* = .079, η_p_^2^ = 0.05). Post-hoc tests revealed that cortisol levels did not significantly change in Tr2 (*p* = .636) and showed a tendency to a decrease in Tr7 (*F*(1.44) = 3.42, *p* = .053, η_p_^2^ = 0.06; Figure 4(f)). Moreover, the main effect of the covariate sex (*F*(1) = 5.31, *p* = .025) as well as the interactions training*sex (*F*(1) = 5.04, *p* = .029, η_p_^2^ = 0.09), time*sex (*F*(1.35) = 5.14, *p* = .017, η_p_^2^ = 0.09), and training*age (*F*(1) = 4.45, *p* = .040, η_p_^2^ = 0.08) were significant. Cortisol levels were significantly higher in men than in women immediately after the cycling training (*t*(55) = 3.49, *p* < .001, *d* = 0.94) and 20 minutes later (*t*(55) = 3.28, *p* = .002, *d* = 0.88) in Tr2 but did not differ at the further measurement time points (all *p* ≥ .58).

### Long-Term Effects

#### Perceived Stress

**Figure 5.**
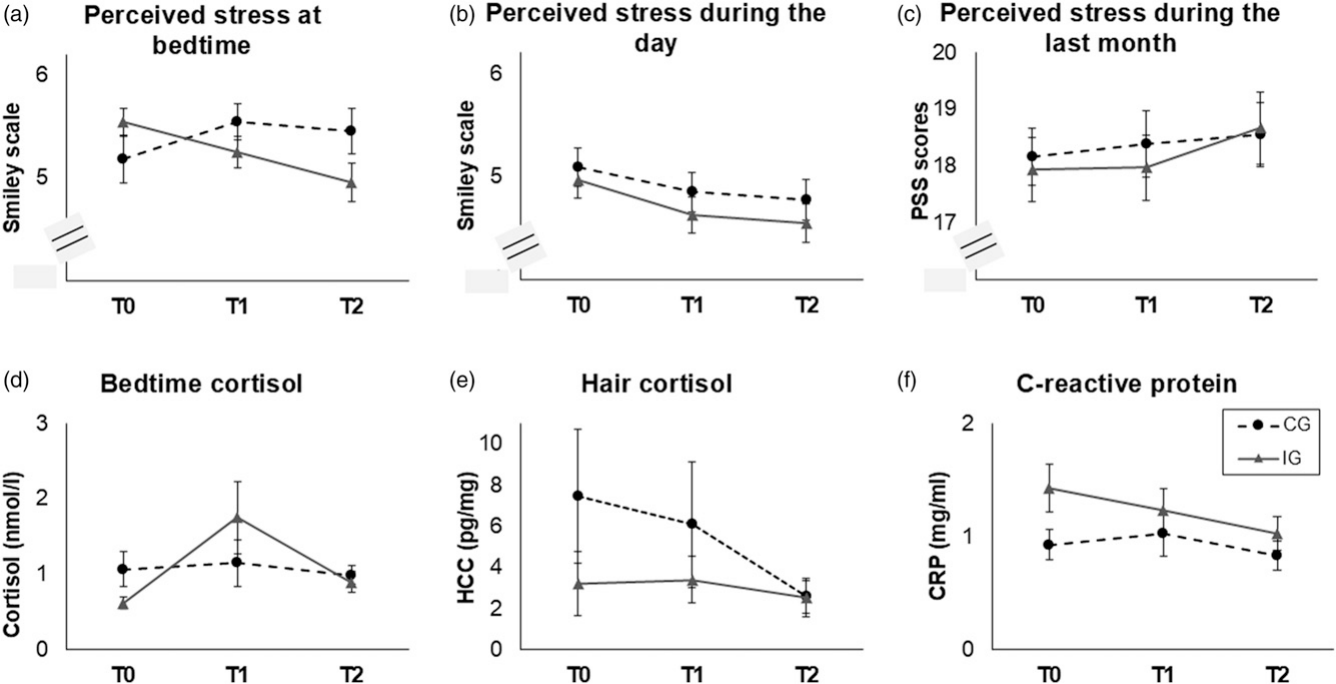
Long-term effects of the cycling intervention in comparison between the intervention group (IG) and the control group (CG) for (a) perceived stress at bedtime during saliva sampling, (b) during the day of saliva sampling, and (c) during the last month, as well as (d) bedtime cortisol levels, (e) hair cortisol concentration (HCC), and (f) C-reactive protein (CRP) levels. Standard errors are shown as error bars. Bedtime cortisol, HCC, and CRP levels were transformed using the natural logarithm for statistical analyses. Note. PSS = Perceived Stress Scale (Cohen et al., 1994; Klein et al., 2016). The range of the smiley scales in (a) and (b) was 1–6.

For the stress ratings at the time point of bedtime cortisol sampling, a significant interaction time*group was found (*F*(1.92) = 4.64, *p* = .013, η_p_^2^ = 0.10), reflecting that perceived stress ratings decreased in the IG (*F*(2) = 6.75, *p* = .003, η_p_^2^ = 0.23) but did not change in the CG (*p* = .338; Figure 5(a)). For the general stress level during the day of bedtime cortisol sampling, a significant main effect of the factor time was found (*F*(2) = 3.45, *p* = .036, η_p_^2^ = 0.07), reflecting a decrease between T0 and T1 in both groups (*t*(45) = 2.05, *p* = .046, *d* = 0.30; Figure 5(b)). For the PSS, no significant effects were found (all *p* ≥ .398; Figure 5(c)).

#### Basal HPA Axis Activity

For bedtime cortisol, a significant interaction time*group was found (*F*(2) = 3.61, *p* = .031, η_p_^2^ = 0.08; Figure 5(d)). Post-hoc tests revealed that cortisol levels did not significantly change in any of the groups (IG: *p* = .320, CG: *p* = .088).

For HCC, a significant main effect of the factor time was found (*F*(2) = 6.33, *p* = .005, η_p_^2^ = 0.27; Figure 5(e)), reflecting an overall decrease in HCC levels between T1 and T2 (*t*(21) = 3.06, *p* = .006, *d* = .65). Furthermore, the interactions between time and the covariates age and BMI were significant (time*age: *F*(2) = 4.50, *p* = .018, η_p_^2^ = 0.21; time*BMI: *F*(2) = 6.83, *p* = .003, η_p_^2^ = 0.29). Post-hoc analyses revealed no significant associations between HCC levels and age or BMI (*p* ≥ .085).

#### Chronic Low-Grade Inflammation

For CRP, only a significant main effect of the covariate sex was found (*F*(1) = 7.31, *p* = .009, η_p_^2^ = 0.13), reflecting lower CRP levels in male than in female participants at all time points (e.g., at T0: *t*(53) = 3.52, *p* < .001, *d* = 0.95; Figure 5(f)).

## Discussion

Our aim was to investigate psychological and physiological stress during a multicomponent cycling intervention in older people. First, we compared acute stress responses during the training sessions Tr2 and Tr7. We found a decrease in acute perceived stress, anxiety, uncertainty, and FoF during both, Tr2 and Tr7. No significant changes were observed for sAA and cortisol.

Second, we analyzed the time course of chronic stress measures (i.e., bedtime cortisol, HCC, and CRP) between three measurement time points T0, T1, and T2 in comparison between the IG and CG. Perceived stress, which was assessed during bedtime saliva sampling, decreased between T0 and T1 in the IG but not in the CG. The long-term stress biomarkers bedtime cortisol, HCC, and CRP did not significantly change between T0, T1, and T2 in any of the groups.

Overall, our study confirms the intervention was associated with reduced perceived acute and chronic stress. Therefore, our findings are in line with previous research that has shown a positive impact of cycling on psychological wellbeing (Bauman & Rissel, 2009; Oja et al., 2011).

However, we did not find the expected changes in biological stress markers. We hypothesized an increase in sAA levels as well as a decrease in cortisol levels during the training sessions, which has been found in previous studies for exercise interventions (e.g., Becker, Semmlinger, & Rohleder, 2021; Kindermann et al., 1982). One reason for this lack of finding may be that the intensity of the acute sessions was too low to induce acute stress responses (Hill et al., 2008). Moreover, sAA baseline levels were high, especially in Tr2. This may be related to the inevitable fact that participants came to the sessions by bike and were thus already engaged in physical activity and their SNS was most likely already activated at the moment of baseline saliva sampling. The time course of cortisol levels pointed in the expected direction (i.e., decreased). Nevertheless, this finding slightly missed statistical significance.

Regarding the acute effects, it must be noted that we did not have a CG during the training sessions. However, since we used established measures for which no changes were found for controls in previous studies (e.g., Becker et al., 2023), it is unlikely that a change would have been found in the current context.

The lack of findings regarding the long-term effects on biological markers is surprising since health benefits of aerobic interventions have been reported numerously (Bourne et al., 2018). Overall, our sample was healthy and participants with baseline levels in the pathophysiological range were excluded from statistical analysis. Therefore, the low baseline levels may have masked significant decreases over time. Moreover, the intervention lasted 8 weeks which may have been too short to trigger long-term physiological adaptation. Furthermore, to the best of our knowledge, our study was the first, in which the specific markers bedtime cortisol, HCC, and high-sensitivity CRP were assessed in the context of a multicomponent cycling intervention in healthy community-dwelling older adults. All these markers are established in research on long-term stress exposure (e.g., Becker, Dupke, & Rohleder, 2021; Kaltenegger et al., 2024; Stalder & Kirschbaum, 2012) but not within our specific context.

Although we only investigated a sub-sample of the original SiFAr trial, we could confirm that the intervention was successful: The number of errors in the cycling course significantly decreased between T0 and T1 in the IG but did not change in the CG. Therefore, our sub-sample was representative. However, our sample included older people with self-perceived needs for improvement in cycling performance. Our findings cannot be generalized to other groups with other motives regarding cycling.

Moreover, the expected positive effects on general FoF and physical performance could also not be found. Again, one reason for this lack of findings may be the low sample size. Furthermore, although SiFAr aimed to be a multicomponent intervention, effects may have not been strong enough to generalize to further domains besides cycling. For health-related quality of life, changes in the expected direction (i.e., an increase between T0 and T1 for the IG) could be observed, which however did not reach statistical significance. Almost half of the participants owned an e-bike and decided for themselves whether they wanted to use the motor during the trainings. Unfortunately, we cannot verify this afterward. However, since the training sessions were of low cardiorespiratory effort overall, the potential use of motors does not interfere with the scope of our study.

A further methodological issue is that some of the participants came to the trainings by bike with a variable distance of biking across subjects, which may have interfered with the baseline measures, particularly with the biological stress markers.

The main limitation of our study is the sample size, which was below the number of 140 participants, who we intended to recruit to achieve a number of 111 participants with complete data for statistical analyses (98/111 = 88%). The reason was the ongoing corona pandemic, which led the main study not achieving its recruitment goal what we were dependent on. Nevertheless, participation rate in our sub-study was good (98/126 = 77.8%). A further limitation is the passive CG. Initially, it was planned that the control group will receive health and bicycle-related presentations in a group setting. Due to the corona pandemic, this had to be cancelled. Therefore, social engagement was one further difference between both groups, which should be taken into account in future research. A further possibility for a future control intervention would be a group walking intervention—possibly complemented by lectures—to achieve a comparable amount of physical activity.

Beside higher sample sizes and a distinct CG, longer durations of the intervention and physically more intense sessions may be needed to find associations with physiological stress markers. Moreover, further measures should be included in future studies which depict how physically demanding the cycling sessions were (e.g., heart rate monitors). Collection of whole blood samples and the assessment of further measures (e.g., interleukin-6) would be another option.

## Conclusions

We conclude that the SiFAr intervention had a positive impact on perceived stress and related concepts and therefore on participants’ wellbeing. In the context of many further previous positive findings such as an increase in mobility and general health, cycling is definitely a recommended means of transportation for community-dwelling older adults. Further research is needed to understand the associations between cycling in older age and physiological stress.
